# Estimates of direct and indirect effects for early juvenile survival in captive populations maintained for conservation purposes: the case of Cuvier's gazelle

**DOI:** 10.1002/ece3.1280

**Published:** 2014-10-10

**Authors:** Belén Ibáñez, Isabel Cervantes, Juan P Gutiérrez, Félix Goyache, Eulalia Moreno

**Affiliations:** 1 Departamento de Ecología Funcional y Evolutiva, Estación Experimental de Zonas Áridas (CSIC)Carretera de Sacramento s/n, La Cañada de San Urbano, Almería, E- 04120, Spain; 2 Departamento de Producción Animal, Universidad Complutense de MadridAvda. Puerta de Hierro s/n, Madrid, E-28040, Spain; 3 Area de Genética y Reproducción Animal, SERIDA-DevaCamino de Rioseco 1225, Gijón, E-33394, Asturias, Spain

**Keywords:** *Gazella cuvieri*, heritability, indirect parental effects, juvenile survival

## Abstract

Together with the avoidance of any negative impact of inbreeding, preservation of genetic variability for life-history traits that could undergo future selective pressure is a major issue in endangered species management programmes. However, most of these programmes ignore that, apart from the direct action of genes on such traits, parents, as contributors of offspring environment, can influence offspring performance through indirect parental effects (when parental genotype and phenotype exerts environmental influences on offspring phenotype independently of additive genetic effects). Using quantitative genetic models, we estimated the additive genetic variance for juvenile survival in a population of the endangered Cuvier's gazelle kept in captivity since 1975. The dataset analyzed included performance recording for 700 calves and a total pedigree of 740 individuals. Results indicated that in this population juvenile survival harbors significant additive genetic variance. The estimates of heritability obtained were in general moderate (0.115–0.457) and not affected by the inclusion of inbreeding in the models. Maternal genetic contribution to juvenile survival seems to be of major importance in this gazelle's population as well. Indirect genetic and indirect environmental effects assigned to mothers (i.e., maternal genetic and maternal permanent environmental effects) roughly explain a quarter of the total variance estimated for the trait analyzed. These findings have major evolutionary consequences for the species as show that offspring phenotypes can evolve strictly through changes in the environment provided by mothers. They are also relevant for the captive breeding programme of the species. To take into account, the contribution that mothers have on offspring phenotype through indirect genetic effects when designing pairing strategies might serve to identify those females with better ability to recruit, and, additionally, to predict reliable responses to selection in the captive population.

## Introduction

Juvenile survival is a critical component of population dynamics. In endangered species managed through captive breeding programmes, the survival of juveniles is crucial for population viability. These conservation programmes focus mainly on the preservation of genetic variability to avoid any negative impact of inbreeding. The genetic effect of inbreeding is the inbreeding depression: the decrease of the individual fitness through reduced fecundity, offspring viability, and individual survivorship (Charlesworth and Charlesworth 1987; Falconer and Mackay 1996). Thus, management of endangered species in captivity tends to minimize mating between relatives to maximize individual fitness and maintain population viability in the long term. This procedure assumes that the improvement of fitness or the threats to fitness are only determined by the probability of individuals carrying identical alleles by descent in a given gene. As neutral markers are assumed to be good indicators for homozygosity, most genetic surveys of endangered populations have been carried out using such molecular tools (Ruiz-López et al. 2009; Godinho et al. 2012) even though they could be poor predictors of genetic diversity in many population scenarios (Hansson and Westerberg 2002).

Undoubtedly, traits of greatest concern in the conservation of evolutionary potential show quantitative variation among individuals (Frankham et al. 2002; Garcia-Gonzalez et al. 2012). Components of quantitative genetic variation determine the ability to undergo adaptive evolution and the effects of inbreeding on reproductive fitness (Frankham et al. 2002). Approaches based on the resemblance of relatives can be used to determine whether endangered populations still show significant additive genetic variation (Falconer and Mackay 1996). Narrow-sense heritability (*h*^*2*^), defined as the proportion of total phenotypic variance that can be ascribed to additive genetic variance (Falconer and Mackay 1996), is the most common within-population measure of genetic diversity used for complex traits (see Charmantier and Garant 2005; Boulding 2008; for reviews). Theory predicts a reduction of heritability after several generations of inbreeding (Falconer and Mackay 1996). Heritability, which determines the evolutionary potential of a quantitative trait (Charmantier and Garant 2005), has been estimated for several life-history traits in wild populations (e.g., Kruuk et al. 2000; Réale and Festa-Bianchet 2000; Wilson et al. 2005; Johnston et al. 2011). However, reports in the literature including estimates of heritability for life-history traits in captive populations of endangered mammals are scant (Pelletier et al. 2009), particularly in ungulates (Ricklefs and Cadena 2008). Juvenile survival, an obvious key life-history trait, has been studied in polygynous mammals, including ungulates. This trait is affected by different factors such as birth weight (Singer et al. 1997), sex (Clutton-Brock et al. 1985), litter composition (Burfening 1972; Ibáñez et al. 2013), maternal characteristics (Pluháček et al. 2007; Ibáñez et al. 2013), demographic parameters (Gaillard et al. 1998), and environmental factors (Singer et al. 1997).

In most breeding programmes of endangered species, approaches for the preservation of genetic variability ignore that apart from heredity, parents, as part of the environment that offspring perceive, can influence their progeny through parental effects. Following Wolf and Wade (2009), parental effects represent the influence of parent's genotype and phenotype to their offspring phenotype, independent of additive genetic effects (Kruuk and Hadfield 2007). When there is variation in the quality of the environment provided by the parents and if that variation reflects genetic differences among individuals, then the environment is partially heritable through the action of these parental effects. These ‘indirect genetic effects’ (*sensu* Wolf et al. 1998) are named indirect because the genes leading to the effects are expressed in the parent, not in the individual whose phenotype is being measured (Garcia-Gonzalez and Simmons 2007). ‘Indirect environmental effects’ (*sensu* Wolf et al. 1998) may also occur when nongenetic (i.e., environmental) influences on the phenotype of one individual (parents) have indirect effects on the phenotype of another individual (offspring; Rositer 1996). The assessment of both genetic and environmental indirect effects has major evolutionary implications and is relevant to captive breeding, as maternal effects include the genetic ability and the nongenetic abilities and strategies available to mothers to influence offspring phenotype, with potentially large-scale demographic results (Mosseu and Fox 1998; Jones 2005; Marshall and Uller 2007; Räsänen and Kruuk 2007).

Information on captive animals is recorded in species-specific databases (called studbooks), representing a wealth of invaluable untapped data for quantitative genetic approaches, as they contain detailed pedigree information rarely available for wild populations (Pelletier et al. 2009). In this study, we used the information recorded in the International Cuvier's Gazelle Studbook to analyze calf survival in the largest captive population of this species, which has been maintained at La Hoya Experimental Field Station (Almería, Spain) for over 35 years. We ran genetic models on this long-term dataset, which while adjusting for systematic environmental effects, took into account the major components of phenotypic variance, the additive genetic component and parental effects. Understanding them and ascertaining their importance to individual fitness requires the implementation of a variance components approach that can separate additive genetic and environmental effects on the phenotype of focal individuals, as they might have evolutionary consequences for the long-term sustainability of the captive population.

*Gazella cuvieri* (Ogilby 1841), a Sahelo-Saharan species, has declined dramatically since the 1950s (Beudels et al. 2005), and only a few small isolated populations seem to remain in its range (Morocco, Tunisia, Algeria), apparently due to excessive hunting, anthropogenic barriers, and habitat degradation (Beudels et al. 2005). Its captive breeding program began at ‘La Hoya’ Experimental Field Station (EEZA-CSIC) in Almería in 1975 from four founders (one male and three females; Moreno and Espeso 2008). For this extremely bottlenecked population, one would expect small additive genetic variation for a life-history trait such as juvenile survival (Price and Schluter 1991), and consequently, (1) a decrease in the response to selection (natural or artificial) for this trait after several generations of inbreeding (Falconer and Mackay 1996) and (2) inbreeding depression, as found by several authors for this fitness trait in this population (Alados and Escós 1991; Cassinello 2005). In this study, we verify these expectations. Moreover, the effect of additive genetic variance on phenotypic variation is compared with the contribution of indirect genetic and environmental effects. We also discuss the relative importance of these two drivers of phenotypic variance for the viability of this captive population of endangered Cuvier's gazelles.

## Material and Methods

### Study population

**Figure 1:**
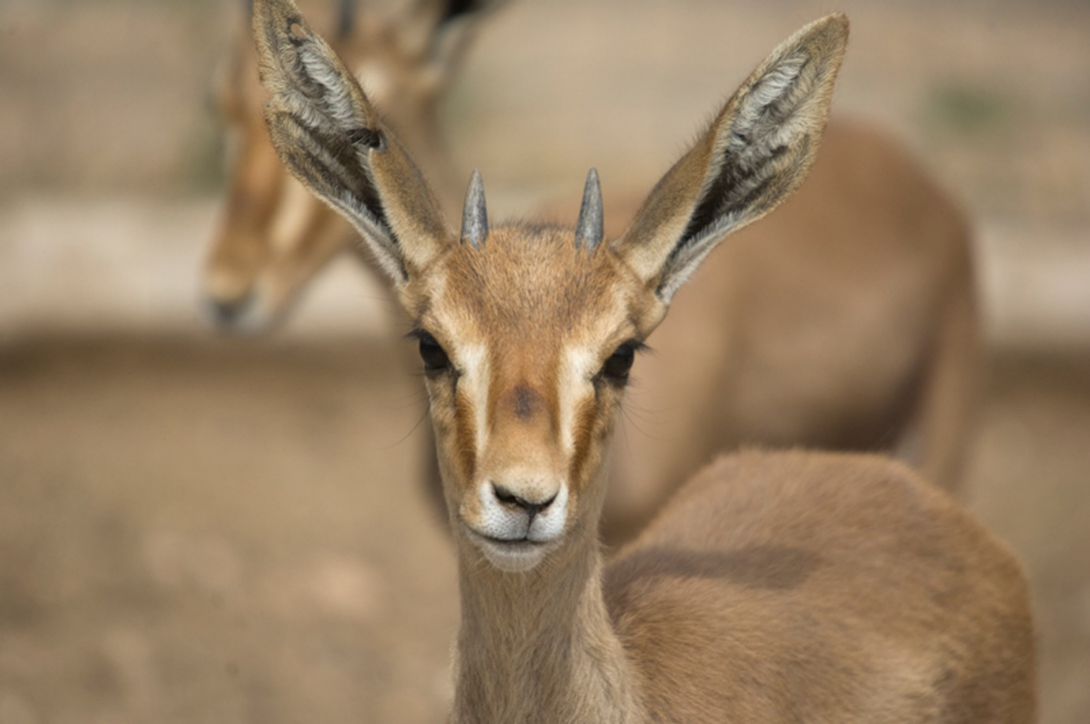
Juvenile of Cuvier's gazelle.

Cuvier's gazelle (Fig. 1) is a medium-sized, sexually dimorphic gazelle. The average body mass of adult females is over 26 kg while that of adult males is about 34 kg. Females are fertile at about 8–9 months and males at 12–13 months. The gestation period is about 5.5 months. Twins represent up to 39% of births in this polygynous species (Moreno and Espeso 2008). At European level, its population is managed through an Endangered Species Programme (EEP) that maintains currently a self-sustaining population. Six institutions (Espeso and Moreno 2012) participate in this EEP, with La Hoya Experimental Field Station (EEZA-CSIC) housing the largest population (currently over 140 individuals). As a general rule, animals at ‘La Hoya’ are maintained in breeding groups formed by one adult male and five to eight adult females. The adult male is removed from its breeding herd when the first calf is born in the herd. This is the recommended procedure in Cuvier's gazelle EEP husbandry guidelines (Moreno and Espeso 2008) to avoid the same male to mate the same females in two consecutive breeding seasons.

Data for the analyses were extracted from the studbook (Espeso and Moreno 2012). Inbreeding coefficient (*F*_*i*_), defined as the probability that an individual has two identical alleles by descent (Wright 1922; Malécot 1948), and individual increase in inbreeding coefficients (Δ*F*_*i*_; Gutiérrez et al. 2008, 2009), defined as the rate to which inbreeding is accumulated in a given individual due to its own pedigree, were calculated from the pedigree in the studbook using the program ENDOG (Gutiérrez and Goyache 2005) which implements the algorithm described by Meuwissen and Luo (1992).

We focus on a critical life-history trait, juvenile survival. In captive populations, as well as in natural ones, the highest mortality occurs among juveniles (Ralls et al. 1979; Kirkwood et al. 1987; Debyser 1995), and in our species mostly up to one month of age (Ibáñez et al. 2013). The trait characterizes the ability of a calf to survive during the period of strict lactation and takes a dichotomous form: live calf (1) and dead calf (0).

Available data were edited to remove records in which calf death was due to management (approximately 0.05% of the total deaths), including traumatisms and injuries due to intraspecific agonistic behavior with adults in the herd. The final dataset analyzed consisted of 700 Cuvier's gazelle calf studbook records (Espeso and Moreno 2012). These included all births at ‘La Hoya’ Experimental Field Station from 1977 to 2012 (an average of 20 offspring per year was recorded). A total of 40 animals without records were included in the pedigree.

### Terminology

The present analysis involves the main following effects:

1. Direct genetic effects (*u*), that is, the variation of a quantitative trait explained by the genotype of the individual on which performance is recorded. Here, the direct genetic effect is referred to calf. The ratio of the variance explained by the direct genetic effect to the total phenotypic variance will be referred as ‘heritability’ (*h*^*2*^).
2. Maternal genetic effects (*m*) defined as any phenotypic influence from a dam on her offspring (excluding the effects of directly transmitted genes) that affect offspring performance (Willham 1963). Biological mechanisms to explain maternal effects include cytoplasmic (mitochondrial) inheritance, intrauterine and postpartum nutrition provided by the dam, antibodies and pathogens transmitted from dam to offspring, and maternal behavior. Due to their genetic nature for dam and their environmental influence for calf, maternal genetic effects are indirect genetic effects. The ratio of the variance explained by the maternal genetic effect to the total phenotypic variance will be referred as ‘heritability of the maternal effect’ (*m*^*2*^).
3. Permanent maternal environmental effects (*c*), that is, those effects on offspring phenotype shared by offspring of the same mother, independent of additive genetic effects. These are a particular case of environmental effects shared by groups of individuals, for instance, effects shared by groups of relatives or individuals belonging to the same cohort. The ratio of the estimates of this effect to the total phenotypic variance will be termed as *c*^2^.

Throughout the text, we use the term ‘systematic’ instead of the term ‘fixed’ to refer to some of the effects included in the models fitted. Although systematic effects are equivalent to those considered fixed in frequentist statistics, in a Bayesian context, where all effects are ‘random’ effects, are not. The difference between ‘systematic’ and ‘random’ effects in a Bayesian context is that the a priori function of the former (that from where the effects of the marginal posterior distribution is sampled) is a flat, uniform function, while the a priori function for random effects is Gaussian.

### Main models

Juvenile survival is a discrete, dichotomous trait. The estimates of genetic parameters in dichotomous traits may depend on the population mean for the trait and, theoretically, threshold models would better account for the probabilistic structure of categorical data than linear models do (Gianola and Foulley 1983; Weller and Gianola 1989). But according to several studies in livestock (Goyache et al. 2003; Cervantes et al. 2010), when databases are small there is little incentive for the use of threshold models over linear models, especially with respect to prediction ability. So in this study, genetic parameters were estimated using a Bayesian procedure applied to linear mixed models (Altarriba et al. 1998), and these models being classified according to the statistical assumptions on the trait as:

1. Continuous (C) model assuming that the analyzed trait was a continuous variable with normal distribution.
2. Threshold (T) model, also called probit, (Gianola 1982; Gianola and Foulley 1983; Sorensen and Gianola 2002) that theoretically would fit the discrete probabilistic nature of the data better. Under this model, it is assumed that an underlying nonobservable variable exists defining the different categories of the categorical trait if this underlying variable exceeds a particular threshold value.

We first analyzed juvenile survival running a complete reference model (equation 1) where offspring survival is treated as a trait of the calf as well as of the mother and of the father; that it, we run a model including all the possible random effects. This model is, however, irresolvable because relationship coefficients involved are less than the number of parameters to be estimated (Hill and Keightley 1988). Its form is given by:


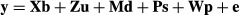
(1)

with


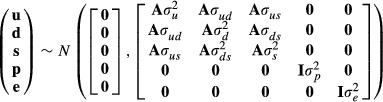


where **y** is the vector of phenotypic measurements of offspring survival; **X** is an incidence matrix relating the values of **y** to the systematic effects parameters given in the vector **b**; **Z** is an incidence matrix relating each of the additive genetic effect to an individual's phenotype, **u** is a vector describing the additive genetic effects; **M** is the incidence matrix of maternal genetic effects (*m*), with **d** as their vector; **P** is the incidence matrix of paternal genetic effects (*s*), with **s** as their vector; **W** is the incidence matrix of maternal permanent environmental effects (*c*), with **p** as their vector; **e** is a vector of residuals effects; 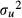 the additive genetic variance, 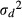 variance due to *m*, 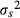 variance due to *s*, *σ*_*ud*_ the covariance between the direct (additive) and the additive genes underlying *m*, *σ*_*us*_ the covariance between the direct (additive) and the additive genes underlying *s*, *σ*_*ds*_ is the covariance between the additive genes underlying *m* and *s*, 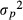 is the variance associated with maternal permanent environmental effects (*c*), **I** is an identity matrix, and **A** is the numerator relationship matrix. Due to the dichotomous nature of the analyzed trait, in threshold models, a restriction was set so that residual variance was set to 1 and threshold was set to 0.

The model includes the following systematic effects in **b**: year of calving (33 levels, from 1977 to 2012; no records available for 1996 because no mating took place in that year; years 2011 and 2012 were pooled since only 4 individuals were born in 2011), mother parity (2 levels: primiparous or multiparous), age of the dam at calving in days, as linear and quadratic covariate, and litter composition [6 levels: F, M, F(F), F(M), M(F), M(M), where M and F mean male and female, respectively, and sibling sex is given in parentheses for twins]. As fitted, this litter composition accounts for the different probability of survival in a male or female twin whether or not the cotwin is the same sex.

In mammals (livestock and wild), the magnitude of maternal effects is generally larger than the magnitude of the paternal effects (Cheverud 1984; Goyache et al. 2003; Wilson and Réale 2006; Blomquist 2012). Thus considering that the above-mentioned model is mathematically irresolvable, and we ran the following alternative models (including fewer random components) where calf survival was treated either as a calf trait or as a combination of calf and mother traits:

1. Calf model: Offspring survival is treated as a trait of calves. In this model, only direct additive genetic effect of the calf is fitted as random effects besides the residual.
2. Calf-dam model: Offspring survival is treated as a trait determined by calves and maternal genetic effects.
3. Calf-permanent model: Offspring survival is treated as a trait determined by calves and maternal permanent environmental effects.
4. Calf-dam-permanent model: Offspring survival is treated as a trait determined by calves, maternal genetic effects, and maternal permanent environmental effects.

These models included 700 calves producing data and a relationship matrix of 740 individuals (Table 1).

**Table 1:** Structure of pedigree used in the Calf model (record for the trait assigned to calves) for the estimation of genetic parameters for juvenile survival in *Gazella cuvieri*

Structure of data	
Number of animals	740
Animals with record	700
Fathers with progeny in data	66
Mothers with progeny in data	196
Fathers with record and offspring	56
Mothers with record and offspring	172
Sire-offspring record pairs	555
Dam-offspring record pairs	612
Year of calving (levels)*	33
Number of primiparous calvings	260
Number of multiparous calvings	440
Number of single calvings	294
Number of twin calvings	460
Number of male calves	356
Number of female calves	344
Average age of mother at calving in years (±SD)	4.26 (2.45)
Average inbreeding of the individuals producing data (±SD)	20.3% (0.07)
Frequency of survival in data	79%

* No records available for year 1996. No calf deaths occurred during 1999 and 2011.

In the studied population, there is no clear evidence for the influence of inbreeding on performance across different life-history traits as some studies have found support for this influence (Alados and Escós 1991; Cassinello 2005), but others not (Ruiz-López et al. 2010; Ibáñez et al. 2013). As inbreeding influence is theoretically defined on nonadditive genetics influence, it is supposed that its effect when fitted as a systematic effect would remove part of the residual variance while keeping the same additive genetic component. Therefore, an increase in heritability would be expected in that scenario. Taking this into account, different models were fitted to ascertain the possible influence of inbreeding on the *Gazella cuvieri* genetic background. Then, models described above were also classified according to the assessment made regarding the influence of inbreeding on the trait as:

Model I: Run without fitting the inbreeding coefficient of the individual producing data in the model.
Model II: Run with the inclusion of the inbreeding coefficient of the individual (F_i_) producing data in the model both as a linear and a quadratic covariate. This model account for the well-known nonlinear relationship between inbreeding coefficients and inbreeding depression (Fernández et al. 2002).
Model III: Run with the inclusion of the individual increase in inbreeding coefficient (ΔF_i_; Gutiérrez et al. 2009) of the individual producing data as a linear covariate. This Model accounts for the stochastic rate of accumulation of inbreeding in each individual along its pedigree, which is theoretically not affected by any nonlinear increase in inbreeding over time (González-Recio et al. 2007; Gutiérrez et al. 2008).

### Complementary models

To acquire further insight into the definitive genetic nature of juvenile survival, the possibility that the trait is only dependent on either the influence of the mother (juvenile survival treated as a mother trait) or the influence of the father (juvenile survival treated as a father trait) should also be explored. Therefore, a number of complementary models were fitted as well to find out the likely influence of the mother, the father, or of both parents in this phenotypic trait of their offspring. A full description of the complementary models fitted, and their results are given in the Supplementary Material and in Tables S1 and S2.

### Statistics

All estimations were carried out in a Bayesian frame using the TM program (Legarra 2008). Marginal posterior distributions of all parameters were estimated using the Gibbs sampling algorithm programmed in TM. In addition, this software enables setting threshold animal models besides continuous models, allowing comparisons between these different models. Prior distributions for vector **b** were assigned as bounded uniform prior distribution, and the variance components 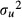, 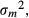 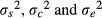 were scaled inverted chi-squared distributions (*v* = 2 and S = 0). A total Gibbs chain length of 1,000,000 samples for each analysis were defined, with a burn-in period of 100,000 and a thinning interval of 100.

Models were tested and examined to choose the one that best predicted performance instead of goodness of fit, as models with the best fit are not always those that provide the best prediction. At present, cross-validation (Efron and Tibshirani 1993) is considered the best method for checking model prediction ability (Arlot and Celisse 2010). As results found when using quantitative models are known to be model dependent as well as database dependent, changes in both the effects included in the model fitted and the size (or structure) of the database analyzed affect predictive power. When the same database is analyzed, a given model may fit better to data. However, when the goal is to predict performance, it must be ensured that the prediction ability of such model does not drop when the database changes. The most common approach to maximizing predictive power is to: (1) Create different random subsets from a given database, (2) Carry out the analyses excluding one of the subsets created, and then (3) Predict the performance of the excluded subset using the results of the analyses. When this ‘cross-validation’ procedure is repeated a number of times for each model, the correlation between the predicted and real performance data can be straightforwardly used to compare models for their prediction ability. The use of cross-validation as the selection criterion has an additional benefit. As this procedure is simply based on the correlation between real (removed) data and the corresponding predicted data, the criterion is free of parametric assumptions. This approach can be applied directly to a wide variety of models with which the predictive power of continuous vs. threshold models can be compared.

To carry out cross-validation, we randomly removed half of the records of the last 5 years of birth (reference population), the genetic parameters reestimated running the models solved without them, and the removed records estimated according to the obtained solutions. The solutions obtained for the records removed were compared to the real performance data via classical correlation to assess the predictive ability of the model. Then, the correlation (r) between the real removed record and the continuous solution (not rounded estimated record in the continuous models and the underlying variable in the threshold models) was computed. To avoid sampling bias, each model was rerun for 20 random samples and the correlation averaged. Once the best model was chosen, additive genetic values were averaged within year of birth to explore signs of genetic trend of the trait.

When the best model had been selected by cross-validation, inferences about systematic effects were carried out in a Bayesian context. Therefore, as marginal posterior distributions are available, inferences can be performed in terms of probability of the parameter being located between arbitrary values. In this case, inferences were provided in terms of probability of some desired parameters being higher than 0.

## Results

### Systematic effects

**Figure 2:**
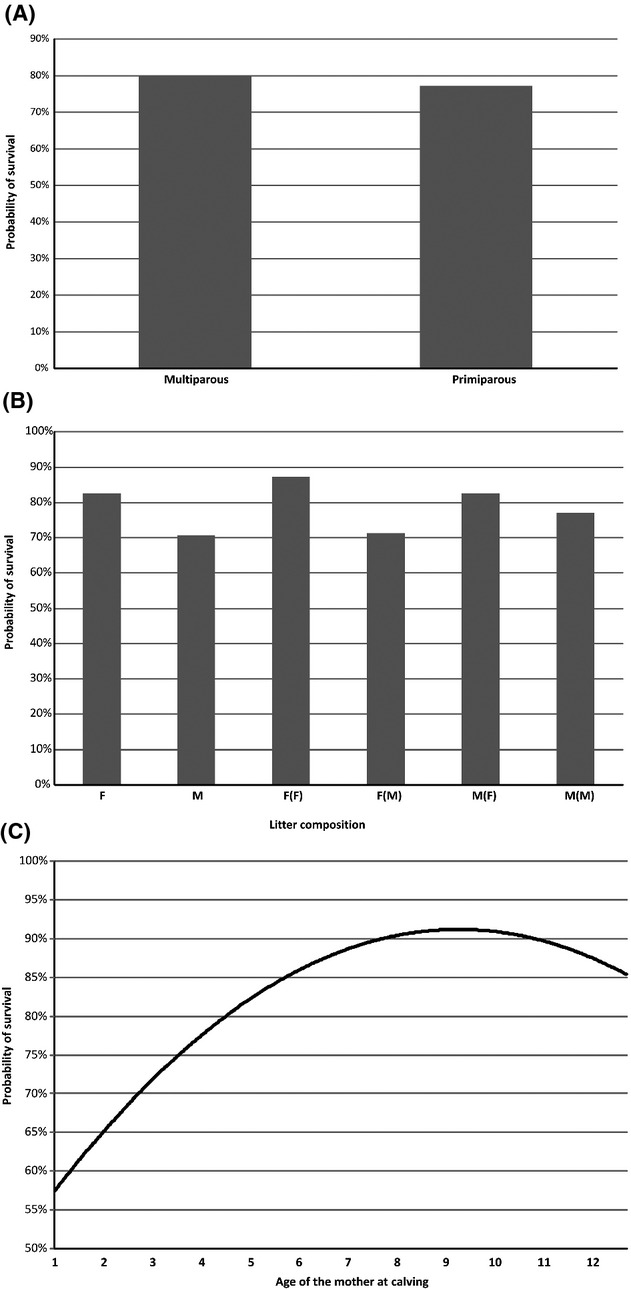
Probability of calf survival considering major systematic effects: mother parity (plot A; primiparous *vs* multiparous), litter composition (Plot B; this factor captures sex and litter size; M and F mean male and female, respectively, and sibling sex is given in parentheses) and mother age (as quadratic covariable) in years (Plot C).

Figure 2 gives information on the solutions found for the major systematic effects included in the linear Calf-dam model. The calf of a multiparous gazelle had four points higher probability of survival than the calves of primiparous gazelles (Fig. 2A), with 79% of probability of being really higher. Male calves had a lower probability of survival than female calves (71% vs. 82%), with 99% of probability of being really lower. When twin females (FF) were compared with twin males (MM), a female still had nine points higher probability of survival (with 95% of probability of being higher). If considering mixed-sex twins, a female with a male cotwin (F(M)) had 13 points lower probability of survival than with a female as a cotwin (FF), with 99% of probability of being lower; however, a male with a female cotwin (M(F)) had 12 points higher probability of survival than with a male as cotwin (MM), with 99% of probability of being higher (Fig. 2B). The age in days of the mother at calving had a positive regression coefficient (0.10 × 10^−3^; 87% of probability being positive) for the linear adjustment and negative (−0.03 × 10^−6^; 87% of probability being negative) for the quadratic adjustment which means that offspring born to young and to old mothers are less likely to survive than those born to middle-aged mothers (Fig. 2C), the optimum of the trait being reached in mothers from 8 to 10 years old.

### Predictive ability

Table 2 gives the mean and standard deviation of the marginal posterior distribution of the parameters estimated for juvenile survival in Cuvier's gazelle using Model I. Under threshold models, the shown parameters were those obtained on the continuous underlying scale. Neither the coefficients of inbreeding (Model II) nor the individual increase in inbreeding (Model III) had relevant effect on the trait analyzed (Appendix S1). When Models II and III were used estimates of the effects included in the models changed less than 3%. Furthermore, the posterior distribution of the differences between the estimates obtained using these Models and Model I always included 0 and, therefore, they could not be considered statistically significant. Therefore, we only give and discuss below results obtained for Model I.

**Table 2:** Mean and standard deviations* (in brackets) of the posterior marginal distribution of the genetic parameters for juvenile survival obtained with the four models run under the assumption of either continuous (continuous model) or categorical (threshold model) nature of the studied trait. Abbreviations: *h*^*2*^, proportion of total phenotypic variance ascribed to additive genetic variance of the individual (calf) producing data (heritability); *m*^2^, proportion of total phenotypic variance ascribed to maternal genetic effects; *c*^2^, proportion of total phenotypic variance attributed to maternal permanent environmental effects; *r*_*g*_, correlation between the genetic components of the effects included in either model fitted; *r*, the mean correlation (20 replicates) between the real removed records and their prediction. Models fitted did not include the inbreeding coefficient of the individual producing data. Residual variance was arbitrarily set to 1 in threshold models

	*h*^2^	*m*^2^	*c*^2^	*r*_*g*_	*r*
Continuous models					
Calf model	0.457 (0.173)				0.061
Calf-dam model	0.359 (0.291)	0.246 (0.237)		0.137 (0.668)	0.103
Calf-permanent model	0.134 (0.113)		0.186 (0.052)	−0.302 (0.649)	0.008
Calf-dam-permanent model	0.305 (0.281)	0.112 (0.135)	0.158 (0.064)	−0.302 (0.649)	0.083
Threshold models					
Calf model	0.245 (0.085)				0.050
Calf-dam model	0.142 (0.097)	0.33 (0.19)		−0.148 (0.682)	0.078
Calf-permanent model	0.067 (0.055)		0.247 (0.067)		0.015
Calf-dam-permanent model	0.115 (0.076)	0.136 (0.124)	0.18 (0.08)	−0.217 (0.658)	0.087

* Standard deviations are given instead of standard errors as results are from Bayesian analyses.

In most cases, the continuous models predicted the data better than their threshold counterparts. The continuous models tended to have a better predictive power (higher *r* values) than their threshold counterparts (Table 2). Heritability estimates of the additive genetic effect found assuming juvenile survival only as a calf trait (Calf model) were higher in the continuous than in the threshold models (*h*^*2*^ = 0.457 ± 0.173 vs. *h*^*2*^ = 0.245 ± 0.0.085). These estimates decreased with inclusion of maternally related random effects in the models fitted (Table 2). In threshold models, estimates of maternal effects (both *m* and *c*) were even higher than estimates of direct additive genetic effects. In continuous models, however, such maternal effects are always lower than direct genetic effects (Table 2). As most estimates correlations (all but Calf-dam continuous model) between the direct effects and maternal effects were negative, they can be considered as nonsignificant taking into account that in all cases the standard deviation of the marginal posterior distribution was very high. The worst predictive power was found for the model considering the influence of the mother solely as environmental (Calf-permanent model; *r* = 0.008 for the continuous and *r* = 0.015 for the threshold model). From all these models, the best prediction ability was shown by the Calf-dam continuous model, with *r* = 0.103 (Table 2). The importance of the genetic background of the mother on the trait was confirmed when complementary models were run (see Tables S1 and S2).

### Genetic trends

**Figure 3:**
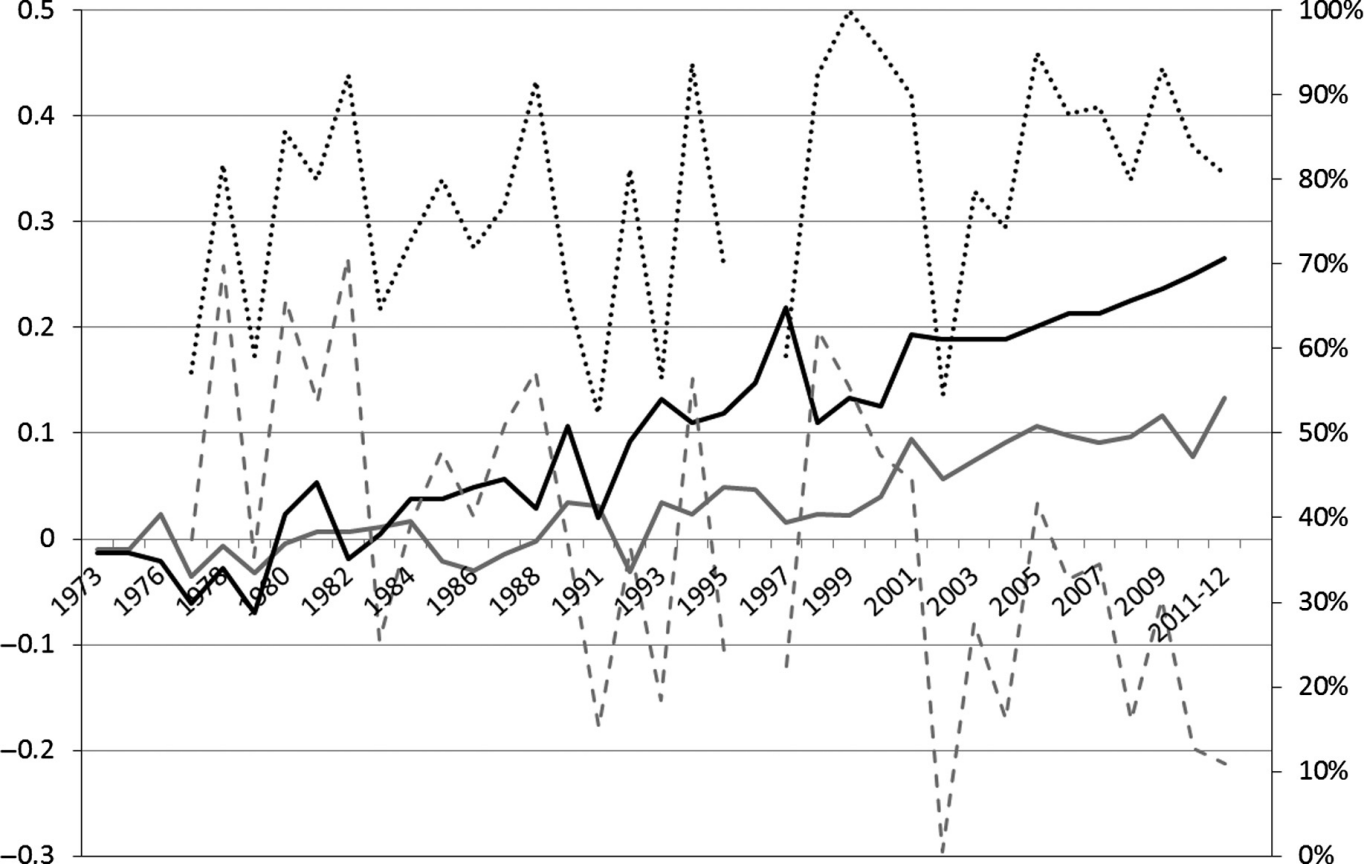
Phenotypic (dotted line, right axis) and mean breeding values of mothers (maternal effect) and individuals (direct genetic effect) in probability of survival by years (black and gray line respectively, left axis) and the year effect (dashed line, right axis) in *Gazella cuvieri*.

Figure 3 shows the phenotypic trend for juvenile survival and the genetic trends for the direct genetic effect estimated using the Calf-dam Model I (which shows the highest *r* value) by year of birth of the individuals. A positive phenotypic trend for juvenile survival over time was found. The genetic ability for juvenile survival has increased over years. The probability of the genetic response to be higher than zero increased across years, increasing from 81% to 89% for the calves and from 71% to 82% for the mothers since 2000). The increase in both calf and mother's genetic ability for the trait was noticeably congruent. As genetic trends were assessed in a Bayesian context, they are not affected by correlated prediction error among cohorts and genetic drift, as they were if we had used the best linear unbiased prediction (BLUP) to predict breeding values (Hadfield et al. 2010).

## Discussion

In this study, we quantified the genetic basis of juvenile survival in a captive population of the endangered Cuvier's gazelle. An understanding of the relative influence of direct (additive genetic) versus indirect (parental) effects underlying this fundamental life-history trait is essential to predict the strength and direction of the evolution of this captive population. In this extremely bottlenecked population, the heritability of juvenile survival is 0.36 (with 98% of probability of being higher than 0.05), which suggests that a non-negligible phenotypic variation observed in this fitness trait is ascribed to additive genetic variance. There are also indirect parental (mainly maternal) effects in this trait which may produce phenotypic resemblance between relatives equivalent to or even greater than that due to the additive genetic variance. Thus, genes influencing juvenile survival are not only those expressed in the individual (directly inherited from calf's parents), but also those of an interacting phenotype, its mother. This means that a calf's phenotype may also evolve through changes in the environment provided by its mother.

### Systematic effects and permanent maternal environmental effects on juvenile survival

Juvenile survival in Cuvier's gazelle is highly influenced by both mother parity and mother age (Fig. 2), which is consistent with results from other nongenetic studies carried out with this (Ibáñez et al. 2013) and other mammal species (Côté and Festa-Bianchet 2001; Pluháček et al. 2007). Offspring survival was relatively low when mothers were young and primiparous (62% at 1 year old), substantially increased when mothers were mid-aged (up to 87% at 8.5 years old) and decreased again in senescent mothers. The optimal age of mothers for calf survival was from 7.5 to 9.5 years old. Breeding before reaching adult body size represents a cost in terms of calf survival added to inexperience on primiparous mothers and decline in offspring survival found in oldest mothers might be the consequence of a decreased body condition due to reproductive senescence (Berubé et al. 1999; Côté and Festa-Bianchet 2001; Ericsson et al. 2001).

Litter composition (a factor that captures sex and litter size) influences infant survival as well in Cuvier's gazelles. The highest mortality was found for single male offspring (M) and for offspring with a male cotwin [F(M); M(M); see also Ibáñez et al. 2013]. Our results in a captive Cuvier's population support findings by other authors that female calves are less costly to produce and rear than males, even if they are twins (Moreno et al. 2011).

Maternal permanent environmental effect also explains a proportion of the variance of juvenile survival. The data fit for the Calf-dam-permanent models were slightly lower than for the Calf-dam models. The small size of the available dataset led to poorer performance of the models fitted as the number of effects included increased. Although these maternal effects do not contribute directly to the evolutionary response to selection (Wolf et al. 1998) they might have important management consequences in captive breeding of threatened species as it might help the EEP's manager to identify those dams providing better environment to their offspring, offering a complementary criteria when arranging breeding herds. For example, the manager might detect those mothers more successful at preventing offspring death because they provide more care, and mate them preferably to others tending more to lose offspring.

### Genetic nature of juvenile survival

Heritability (*h*^*2*^) of juvenile survival in the Cuvier's gazelle was moderate (Table 2), but much higher than estimates of *h*^*2*^ in captive rhesus macaques (Gagliardi et al. 2010). It was also higher than estimates of *h*^*2*^ for other life-history traits in wild red deer (Kruuk et al. 2000) and other mammals (Holt et al. 2005). Contrary to expectations, our results suggest that some significant amount of additive genetic variance is maintained within this captive population for a character closely related to fitness, revealing that this quantitative trait can potentially still evolve (Charmantier and Garant 2005). Moreover, we found that heritability estimates (*h*^*2*^) were higher when the trait was considered only as a calf trait than with the inclusion of maternally related random effects in the models fitted, suggesting that the additive genetic variances were overestimated due to previously unaccounted for genetic and environmental maternal effects. In our analyses, the maternal variance components indicated that mothers vary in their influence on the survival of their offspring. The models fitted allowed us to separate maternal variance from offspring additive variance. As maternal effects were consistent across models, we infer that indirect maternal effects operate on juvenile survival through maternal selection. When maternal genetic effects are not negligible, response to selection depends not only on direct, but also on the additive genes underlying the maternal genetic effect (*m*), which can result in accelerated, or dampened response to selection (Wolf et al. 1998). Here, looking at the standard deviations of its posterior marginal distribution, the genetic correlation estimated between *u* and *m* was clearly nonsignificant regardless of the model used. Hence, the use of individual additive genetic values for survival as criteria to form breeding herds in this captive population will make sense only if the maternal genetic effects are considered. By doing this, juvenile mortality will tend to decrease in the population thereby increasing its long-term viability.

A positive change in genetic trend was thus observed in calves and mothers, which shows selection for juvenile survival over time. These results indicate that (1) the Cuvier's Gazelle captive breeding program is effective in achieving genetic improvement in this fitness trait despite increased inbreeding since it began in 1975 (Ibáñez et al. 2011) and (2) that genetic changes have occurred in response to natural selection attesting to the evolutionary potential of this captive population.

### Influence of inbreeding

The inclusion of inbreeding in the estimation models (Appendix S1) did not affect the estimates of heritability, suggesting the maintenance of genetic variability in our population. Although a potential change in variance components dependant on inbreeding has not been modelled, if such relationship exists, residual variance would have decreased and heritability would have increased. Even when inbreeding increased, there was no depression, as juvenile survival progressively increased over the 35-year study period. The low impact of inbreeding depression observed in our study (see also Ibáñez et al. 2011, 2013) could be a consequence of a slow rate of inbreeding in the Cuvier's gazelle population in the past, which may have allowed natural selection to progressively purge some of the negative consequences of inbreeding (Ballou 1997), or it could just be a specific feature of the species, where the consequences of inbreeding seem to be less striking than in others (Ballou 1994). Improvements in husbandry may lead to higher average survival in captive populations in spite of an increase in inbreeding as well (Kalinowski et al. 1999). Although we cannot exclude this possibility, the importance of maternal effects suggests that the increase in calf survival is not solely due to husbandry improvements.

## Insights for conservation

For threatened and endangered species, coordinated captive breeding programs such as the European Endangered Species Programme (EEP) represent the only way to rear and maintain the sustained populations that ensure their survival (Magin et al. 1994; Russello and Amato 2004). However, captive breeding populations are also often observed to be in serious demographic decline. Although their managers have a variety of breeding schemes for maintaining their genetic diversity and alleviating inbreeding depression if necessary, achieving sustainable population sizes of these generally low-founder populations is usually difficult (Kleiman et al. 2010). In this study, we have focused on a key fitness trait, juvenile survival, which represents the greatest contribution to fitness in both captive and natural populations (Houde et al. 2013). Our results underscore that, apart from direct genetic transmission, parents (mainly mothers) contribute to their offspring through indirect (genetic and environmental) effects, these maternal effects increasing the potential of this population to respond to selection on offspring survival. So, to take into account maternal contribution in pairing strategies of captive bred endangered species might be of great importance in predicting a reliable response to selection, as well as to identify those individuals with better ability to recruit. Even more, if traits expressed during social interactions (e. g., mother–offspring interaction) evolved more rapidly than other type of traits (Moore et al. 1997), to consider their likely effects is crucial when arranging pairing strategies as they might be responsible at least partially for the rapid adaptation to captivity described for some species (Frankham and Loebel 1992; Woodworth et al. 2002; Heath et al. 2003; Kraaijeveld-Smit et al. 2006).

## Supporting Information

**Appendix S1.** Mean and standard deviations (in brackets) of the posterior marginal distribution of the genetic parameters for juvenile survival in a captive population of *Gazella cuvieri*.

**Table S1.** Number of animals with record and structure of pedigree used for the estimation of genetic parameters for juvenile survival in *Gazella cuvieri*. **Table S2.** Mean and standard deviations (in brackets) of the posterior marginal distribution of the genetic parameters for juvenile survival obtained with the four models run under the assumption of either continuous or categorical (threshold) nature of the studied trait.
