# Supplementary material for: Estimates of direct and indirect effects for early juvenile survival in captive populations maintained for conservation purposes: the case of Cuvier's gazelle
Source: Ecol Evol. 2014 Oct 10;4(21):4117–29. doi: 10.1002/ece3.1280 (PMC4242564; doi:10.1002/ece3.1280)
Supplement: Supplementary file 1 — Appendix S1. Mean and standard deviations (in brackets) of the posterior marginal distribution of the genetic parameters for juvenile survival in a captive population of Gazella cuvieri. [file ece30004-4117-SD1.docx]

**Appendix**

|  | **Continuous models** | | | | | **Threshold models** | | | | |
| --- | --- | --- | --- | --- | --- | --- | --- | --- | --- | --- |
| Model II^a^ | h^2^ | *c*^2^ | *m*^2^ | *s*^2^ | r_g_ | h^2^ | *c*^2^ | *m*^2^ | *s*^2^ | r_g_ |
| Calf model | 0.457 (0.192) |  |  |  |  | 0.241 (0.086) |  |  |  |  |
| Calf-dam model | 0.424 (0.501) |  | 0.244 (0.350) |  | 0.110 (0.681) | 0.169 (0.116) |  | 0.353 (0.209) |  | -0.302 (0.646) |
| Calf-permanent model | 0.122 (0.107) | 0.188 (0.051) |  |  |  | 0.066 (0.056) | 0.249 (0.068) |  |  |  |
| Calf-dam-permanent model | 0.311 (0.278) | 0.155 (0.064) | 0.109 (0.124) |  | -0.327 (0.693) | 0.116 (0.079) | 0.176 (0.078) | 0.143 (0.126) |  | -0.314 (0.651) |
| Dam model | 0.228 (0.070) |  |  |  |  | 0.296 (0.081) |  |  |  |  |
| Dam-permanent model | 0.070 (0.050) | 0.160 (0.050) |  |  |  | 0.110 (0.080) | 0.200 (0.080) |  |  |  |
| Sire model |  |  |  | 0.038 (0.031) |  |  |  |  | 0.094 (0.064) |  |
| Dam-sire model |  |  | 0.261 (0.078) | 0.031 (0.029) | -0.191 (0.672) |  |  | 0.339 (0.104) | 0.062 (0.059) | -0.197 (0.664) |
| Model III^b^ |  |  |  |  |  |  |  |  |  |  |
| Calf model | 0.453 (0.187) |  |  |  |  | 0.240 (0.085) |  |  |  |  |
| Calf-dam model | 0.385 (0.347) |  | 0.242 (0.276) |  | 0.156 (0.699) | 0.158 (0.103) |  | 0.341 (0.196) |  | -0.272 (0.664) |
| Calf-permanent model | 0.122 (0.077) | 0.166 (0.047) |  |  |  | 0.089 (0.059) | 0.217 (0.066) |  |  |  |
| Calf-dam-permanent model | 0.329 (0.283) | 0.149 (0.065) | 0.110 (0.134) |  | -0.288 (0.682) | 0.116 (0.079) | 0.176 (0.078) | 0.143 (0.126) |  | -0.314 (0.651) |
| Dam model | 0.230 (0.069) |  |  |  |  | 0.293 (0.082) |  |  |  |  |
| dam-permanent model | 0.060 (0.050) | 0.170 (0.060) |  |  |  | 0.110 (0.080) | 0.200 (0.080) |  |  |  |
| Sire model |  |  |  | 0.037 (0.030) |  |  |  |  | 0.095 (0.065) |  |
| Dam-sire model |  |  | 0.266 (0.083) | 0.033 (0.035) | -0.206 (0.693) |  |  | 0.332 (0.103) | 0.056 (0.054) | -0.172 (0.671) |

^a^ Model II was fitted including the inbreeding coefficient of the individual producing data in the model both as a linear and a quadratic covariate.

^b^ Model III was fitted including in the model the individual increase in inbreeding coefficient of the individual producing data as a linear covariate.

**Appendix legend**

Mean and standard deviations (in brackets) of the posterior marginal distribution of the genetic parameters for juvenile survival in a captive population of *Gazella cuvieri*. Estimates were obtained using different models run under the assumption of either continuous (continuous model) or categorical (threshold model) nature of the studied trait and including the inbreeding coefficient of the individual producing data in the model both as a linear and a quadratic covariate (Model II) or the individual increase in inbreeding coefficient of the individual producing data as a linear covariate (Model III). Abbreviations: *h^2^*, proportion of total phenotypic variance ascribed to additive genetic variance of the individual (calf) producing data (heritability); *c*^2^, proportion of total phenotypic variance attributed to maternal permanent environmental effects; *m*^2^, proportion of total phenotypic variance ascribed to maternal genetic effects; *s*^2^, proportion of total phenotypic variance ascribed to paternal genetic effects; r_g_, correlation between the genetic components of the effects included in either model fitted. Models fitted did not include the inbreeding coefficient of the individual producing data. Residual variance was arbitrarily set to 1 in threshold models.
