# Supplementary material for: Estimates of direct and indirect effects for early juvenile survival in captive populations maintained for conservation purposes: the case of Cuvier's gazelle
Source: Ecol Evol. 2014 Oct 10;4(21):4117–29. doi: 10.1002/ece3.1280 (PMC4242564; doi:10.1002/ece3.1280)
Supplement: Supplementary file 2 — Table S1. Number of animals with record and structure of pedigree used for the estimation of genetic parameters for juvenile survival in Gazella cuvieri. Table S2. Mean and standard deviations (in brackets) of the posterior marginal distribution of the genetic parameters for juvenile survival obtained with the four models run under the assumption of either continuous or categorical (threshold) nature of the studied trait. [file ece30004-4117-SD2.docx]

**SUPLEMENTARY MATERIAL**

To know how important maternal and paternal effects are for juvenile survival in our population we have run a number of models where the trait is treated as either a mother or a father or a mother-father trait. Although the effects of the mother on offspring phenotype are expected to be larger than that of the father and paternal genetic effects have been largely dismissed in species where males are not involved in extended parental care, they might explain variations in embryo viability (Garcia-Gonzalez and Simmons 2007), female reproductive effort (De Lope and Möller 1993) and brood sex-ratio (Svensson and Nilsson 1996). Thus, the following models were run:

1. Dam model: offspring survival is treated as a trait determined by maternal genetic effects. Note that given that no direct genetic effect of the calf is included, the dam effect will gather all the maternal effect plus half the additive genetic effect of the calf if exists. This confusion will be affecting all the tested models ignoring the calf.
2. Dam-permanent model: offspring survival is treated as a trait determined by maternal genetic effects and maternal permanent environmental effects.
3. Sire model: offspring survival is treated as a trait determined by paternal genetic effects. We defined “paternal genetic effect” (s) as any phenotypic influence from a sire on his offspring (excluding the effects of directly transmitted genes) that affect offspring performance. The ratio of the variance explained by the paternal genetic effect to the total phenotypic variance will be referred as “heritability of the paternal effect” (s^2^).
4. Dam-Sire model: offspring survival is treated as a trait determined by maternal and paternal genetic effects.

Genetic parameters were estimated using a Bayesian procedure applied to mixed linear models, they being classified according to the statistical assumption of the trait as either “continuous” or “threshold” models (see more details in the main text: Material and Method section).

The Dam model included 196 mothers producing data and a total pedigree of 254 individuals; the Sire model included 66 fathers producing data and a total of 139 individuals in the pedigree matrix; and the Dam-Sire model included 262 animals producing data and 268 individuals in the pedigree matrix (Table 1S). Table 2S gives the mean and the standard deviation of the posterior distribution of the parameters estimated for juvenile survival in Cuvier’s gazelle under the different assumptions for individual producing data. Under the threshold models the shown parameters are those obtained on the continuous underlying scale. As for the Main models, neither the coefficients of inbreeding nor the individual increase in inbreeding had a relevant effect on the trait analysed (see Appendix).

Results show that if offspring survival is treated as a trait determined solely by maternal genetic effects, models run showed heritability estimates of 0.253 ± 0.071 and 0.311 ±0.081 for continuous and threshold models, respectively. These figures were even higher than those estimated for the maternal effect (*m*) using the Calf-dam continuous model. This suggest that if the own genetic ability of the calf to survive is neglected, the estimates of the importance of the mother on this trait is biased upwards. In any case, the estimates of *m^2^* fell to 0.070 ± 0.056 and 0.114 ± 0.085 (Table 2S) when maternal permanent environmental effects were included in the models which points to the relevance of this indirect environmental effect in disentangling the variance components of juvenile survival in Cuvier’s gazelle.

When compared with the Dam models the fit with data of the Sire models was poor (Table 2S), suggesting that the paternal influence on the trait was of limited importance. The estimates of the paternal genetic effect *s^2^* were very low (roughly 3%) assuming continuous variation of the trait, and low for the threshold models (0.091 ± 0.062 and 0.053 ± 0.053 for the Sire and the Dam-sire models respectively; Table 2S). On the contrary, the heritability estimates of the maternal genetic effect using the Dam-sire model were moderate (0.288 ± 0.083 and 0.346 ± 0.105 for the continuous and threshold models, respectively; Table 2S). The genetic correlation between *m* and *s* was moderate and negative. Moreover, the marginal posterior distribution for this correlation showed high standard deviation; this would suggest that the paternal and maternal genetic background influencing calf survival would not be the same.

Maternal genetic contribution to juvenile survival seems to be of major importance in the studied Cuvier’s gazelle population. Theoretically, when using the Dam model the estimates of the variance ascribed to maternal genetic effect (*m^2^*) would include the additive genes of the mother affecting juvenile survival but also a quarter of the variance associated to the additive genes assignable to the calf. However, the estimates of *m^2^* were much higher than expected under the assumption of complete calf determination of the trait (compare Table 2 and Table 2S). One could think that this is an artefact causing upward bias in the two variances due to the difficulties found for the estimation of the covariance between the direct additive genetic effect of the calf and the additive genetic background underlying the maternal genetic effects. Actually, genetic variance on which selection acts (through juvenile viability) is a consequence of both additive and maternal genetic variances and the covariance between them. This association leaded to Willham (1972) to define the total heritability as the sum of the total additive genetic variance, half the maternal genetic variance and one and a half the covariance. These covariances were negative and high, as usually found in livestock populations (Cubas et al. 1989; Cervantes et al. 2010), therefore tending to inflate the estimates of both *h^2^* and *m^2^* (Gutiérrez et al. 1997; Meyer 1997). This is why the maternal contribution to the trait was indirectly assessed using the Dam-sire model. Again the contribution of the mother to the determination of the trait was high, particularly considering the very low estimates for the influence of the father on the trait. Although a negative correlation between the additive genes underlying paternal *(s)* and maternal *(m)* effects may also inflate the variances assigned to the mother effect, it is clear that in this population the contribution of the mother is of major importance for the genetic determination of the trait.

**Literature cited**

Garcia-Gonzalez F, Simmons LW (2007) Paternal indirect genetic effects on offspring viability and the benefits of polyandry. Current Biology 17: 32-36.

De Lope F, Möller A (1993) Female reproductive effort depends on the degree of ornamentation of their mates. Evolution 47: 1152-1160.

Svensson E, Nilsson JA (1996) Mate quality affects offspring sex ratio in blue tits. Proc. R Soc Lond (B) 263: 357-361.

Willham RL (1972) The role of maternal effects in animal breeding: III Biometrical aspects of maternal effects in animals. J Anim Sci 35:1288-1293.

Cubas AC, Berger PJ, Healey MH (1989) Genetic parameters for calving ease and survival at birth in Angus field data. J Anim Sc 69: 3952–3958.

Cervantes I, Gutiérrez JP, Fernández I, Goyache F (2010) Genetic relationships among calving ease, gestation length, and calf survival to weaning in the Asturiana de los Valles beef cattle breed. J Anim Sci 88: 96-101.

Gutiérrez JP, Cañón J, Goyache F (1997) Estimation of direct and maternal genetic parameters for preweaning traits in the Asturiana de los Valles beef cattle breed through animal and sire models. J Anim Breed Genet 114: 261–266.

Meyer K (1997) Estimates of genetic parameters for weaning weight of beef cattle accounting for direct-maternal environmental covariances. Livest Prod Sci 52: 187–199.

Table 1S. Number of animals with record and structure of pedigree used for the estimation of genetic parameters for juvenile survival in *Gazella cuvieri*. Dam model: record for the trait is assigned to calf’s mother; Sire model: record for the trait is assigned to calf’s father; Dam-sire model: record for the trait is assigned to calf’s parents.

| Structure of data | Dam model | Sire model | Dam-sire model |
| --- | --- | --- | --- |
| Number of animals | 254 | 139 | 268 |
| Animals with record | 196 | 66 | 262 |
| Fathers with progeny in data | 52 | 29 | 81 |
| Mothers with progeny in data | 94 | 47 | 141 |
| Fathers with record and offspring | - | 28 | 28 |
| Mothers with record and offspring | 92 | - | 92 |
| Sire-offspring record pairs | - | 639 | 639 |
| Dam-offspring record pairs | 639 | - | 639 |
| Year of calving (levels)^a^ | 33 | 33 | 33 |
| Number of primiparous calvings | 260 | 260 | 260 |
| Number of multiparous calvings | 440 | 440 | 440 |
| Number of single calvings | 294 | 294 | 294 |
| Number of twin calvings | 460 | 460 | 460 |
| Number of male calves | 356 | 356 | 356 |
| Number of female calves | 344 | 344 | 344 |
| Average age of mother at calving in years (± s.d.) | 4.26 (2.45) | 4.26 (2.45) | 4.26 (2.45) |
| Average inbreeding of the individuals producing data (± s.d.) | 18.8% (0.08) | 16.5% (0.08) | 18.2% (0.08) |
| Frequency of survival in data | 79% | 79% | 79% |

^a^ *No records available for year 1996. No calf deaths occurred during 1999 and 2011.*

Table 2S. Mean and standard deviations (in brackets) of the posterior marginal distribution of the genetic parameters for juvenile survival obtained with the four models run under the assumption of either continuous or categorical (threshold) nature of the studied trait. Abbreviations: *c*^2^, proportion of total phenotypic variance attributed to maternal permanent environmental effects; *m*^2^, proportion of total phenotypic variance ascribed to maternal genetic effects; *s^2^*, proportion of total phenotypic variance ascribed to paternal genetic effects; r_g_, correlation between the genetic components of the effects included in either model fitted. Models fitted did not include the inbreeding coefficient of the individual producing data. Residual variance was arbitrarily set to 1 in threshold models.

|  | Continuous models | | | | | Threshold models | | | | |
| --- | --- | --- | --- | --- | --- | --- | --- | --- | --- | --- |
|  |  | *c*^2^ | *m*^2^ | *s*^2^ | r_g_ |  | *c*^2^ | *m*^2^ | *s*^2^ | r_g_ |
| Dam model |  |  | 0.253 (0.071) |  |  |  |  | 0.311 (0.081) |  |  |
| Dam-permanent model |  | 0.170 (0.050) | 0.070 (0.056) |  |  |  | 0.220 (0.081) | 0.114 (0.085) |  |  |
| Sire model |  |  |  | 0.036 (0.028) |  |  |  |  | 0.091 (0.062) |  |
| Dam-sire model |  |  | 0.288 (0.082) | 0.033 (0.031) | -0.169 (0.693) |  |  | 0.346 (0.105) | 0.053 (0.053) | -0.136 (0.689) |
